# Cytotoxicity of Carbon Nanotubes, Graphene, Fullerenes, and Dots

**DOI:** 10.3390/nano13091458

**Published:** 2023-04-25

**Authors:** Marianna V. Kharlamova, Christian Kramberger

**Affiliations:** 1 Centre for Advanced Materials Application (CEMEA), Slovak Academy of Sciences, Dúbravská cesta 5807/9, 845 11 Bratislava, Slovakia; 2 Faculty of Physics, University of Vienna, Boltzmanngasse 5, 1090 Vienna, Austria

**Keywords:** cytotoxicity, carbon nanotube, graphene, fullerene, carbon dots

## Abstract

The cytotoxicity of carbon nanomaterials is a very important issue for microorganisms, animals, and humans. Here, we discuss the issues of cytotoxicity of carbon nanomaterials, carbon nanotubes, graphene, fullerene, and dots. Cytotoxicity issues, such as cell viability and drug release, are considered. The main part of the review is dedicated to important cell viability issues. They are presented for A549 human melanoma, *E. coli*, osteosarcoma, U2-OS, SAOS-2, MG63, U87, and U118 cell lines. Then, important drug release issues are discussed. Bioimaging results are shown here to illustrate the use of carbon derivatives as markers in any type of imaging used in vivo/in vitro. Finally, perspectives of the field are presented. The important issue is single-cell viability. It can allow a correlation of the functionality of organelles of single cells with the development of cancer. Such organelles are mitochondria, nuclei, vacuoles, and reticulum. It allows for finding biochemical evidence of cancer prevention in single cells. The development of investigation methods for single-cell level detection of viability stimulates the cytotoxicity investigative field. The development of single-cell microscopy is needed to improve the resolution and accuracy of investigations. The importance of cytotoxicity is drug release. It is important to control the amount of drug that is released. This is performed with pH, temperature, and electric stimulation. Further development of drug loading and bioimaging is important to decrease the cytotoxicity of carbon nanomaterials. We hope that this review is useful for researchers from all disciplines across the world.

## 1. Introduction

The carbon nanomaterials, such as carbon nanotubes (CNTs), graphene, fullerenes, and dots, attract the attention of researchers because of their unique physical properties. Carbon nanomaterials can cause damage and intoxication to organisms. The problem of toxicity of carbon nanomaterials for plants, microorganisms, animals, humans, and the natural environment is very important. Regarding humans, people work in laboratories worldwide in intercultural environments, and it is an important issue to save people from toxic substances. 

The toxicology of carbon nanomaterials was studied for carbon nanotubes [1,2,3,4,5,6,7,8,9,10,11,12,13,14], graphene [15,16,17,18,19,20,21,22,23,24,25,26,27,28], fullerenes [29,30,31,32,33], and dots [34,35,36,37,38]. Most of the work deals with microorganisms, animals, and humans. The toxicology issues are connected with drug delivery and bioimaging. The drug delivery is controlled by water solubility, biocompatibility, blood circulation time, targeting, and accumulation of therapeutics in diseased cells and tissues for carbon nanotubes [39,40,41,42,43,44,45,46,47,48,49], graphene [50,51,52,53,54,55,56,57,58,59,60,61,62], fullerenes [63], dots [64,65,66,67]. The diagnostic method, bioimaging, is connected with the penetration of drugs and diagnostic agents into tissues and cells and the sensitivity of detection. Bioimaging is controlled by biodegradability, biocompatibility, specificity, and applicability for carbon nanotubes [68,69,70,71,72,73,74,75,76,77,78,79,80,81], graphene [82,83,84,85,86,87,88,89,90,91,92,93,94], fullerenes [95], and dots [96].

These problems can be resolved by the chemical functionalization of carbon nanomaterials. There are five ways to chemically functionalize carbon nanomaterials. Among them is the covalent functionalization of the outer surface of carbon nanotubes [97,98,99,100], noncovalent functionalization of the outer surface of carbon nanotubes [101,102], the substitution of carbon atoms with other atoms [103,104,105,106,107,108,109,110,111,112,113,114,115,116,117,118,119,120,121,122,123,124,125], intercalation of carbon nanotube bundles [126,127,128,129,130,131,132,133,134,135,136,137,138,139,140,141,142,143,144,145,146,147,148,149,150,151,152], and filling of carbon nanotubes [153,154]. 

The covalent functionalization is performed via the covalent bonds between the functional groups and carbon nanomaterials. The functional groups are further used for the bioconjugation of carbon nanomaterials with therapeutic and targeting agents. This decreases the toxicity of carbon nanomaterials and increases the selectivity of drug delivery and the sensitivity and accuracy of bioimaging [155,156]. The noncovalent functionalization is performed via hydrophobic interactions, π-π interactions, and van der Waals forces between carbon nanomaterials and guest molecules. The functional groups can further be conjugated with therapeutic agents. The substitution and intercalation of carbon nanomaterials are other promising ways to functionalize them for theranostic applications. Endohedral chemical functionalization (filling of carbon nanotubes) is an important method for drug delivery and bioimaging applications. It is a very simple and viable method of chemical functionalization for biomedical applications. The filling ratios of medicines are controlled with the synthesis procedures. 

In this review, we consider the functionalization methods of carbon nanomaterials and the cytotoxicity of carbon nanotubes, graphene, fullerene, and dots. In Section 3, we consider the microscopy issues. In Section 4, we highlight the theoretical methods. In Section 5, we discuss cell viability issues. In Section 6, we describe the drug release issues. In Section 7, we introduce perspectives. 

## 2. Cytotoxicity Issues

There are three main issues of cytotoxicity:

- Materials characterization, and theoretical considerations,
- Cell viability,
- Drug release.

For cytotoxicity issues, it is important to introduce the material characterization methods, such as microscopy, spectroscopy (Raman spectroscopy, optical absorption spectroscopy (OAS), Fourier transformed infrared spectroscopy (FT-IR)), and zeta potential measurements. There is a number of reports on the characterization of the material. We chose the most important reports to review here. 

Theoretical works exist for carbon nanotubes, graphene, and fullerenes. Authors consider drug delivery systems with fullerenes and carbon nanotubes with defined atomic structures. The peculiarities of the drug delivery system and drug release are considered. Important structural models of drug delivery systems of such drugs as doxorubicin (DOX) are calculated. These issues are highlighted here.

The main part of the review is dedicated to the discussion of cytotoxicity studies. They are presented for A549 human melanoma cells, *E. coli*, osteosarcoma cell lines, the U2-OS cell line, the SAOS-2 cell line, the MG63 cell line, the U87 cell line, and the U118 cell line. The results are shown in bar diagrams, which are supported by images of cells and bacteria cultured with carbon nanomaterials. 

The next important part of the review is drug release. The results are shown in drug release plots versus time under different pH for different functionalized carbon nanomaterials. Bioimaging results are shown here to illustrate the use of carbon derivatives as markers in any type of imaging used in vivo/in vitro. The review finishes with perspectives. 

## 3. Material Characterization

### 3.1. Microscopy

**Figure 1:**
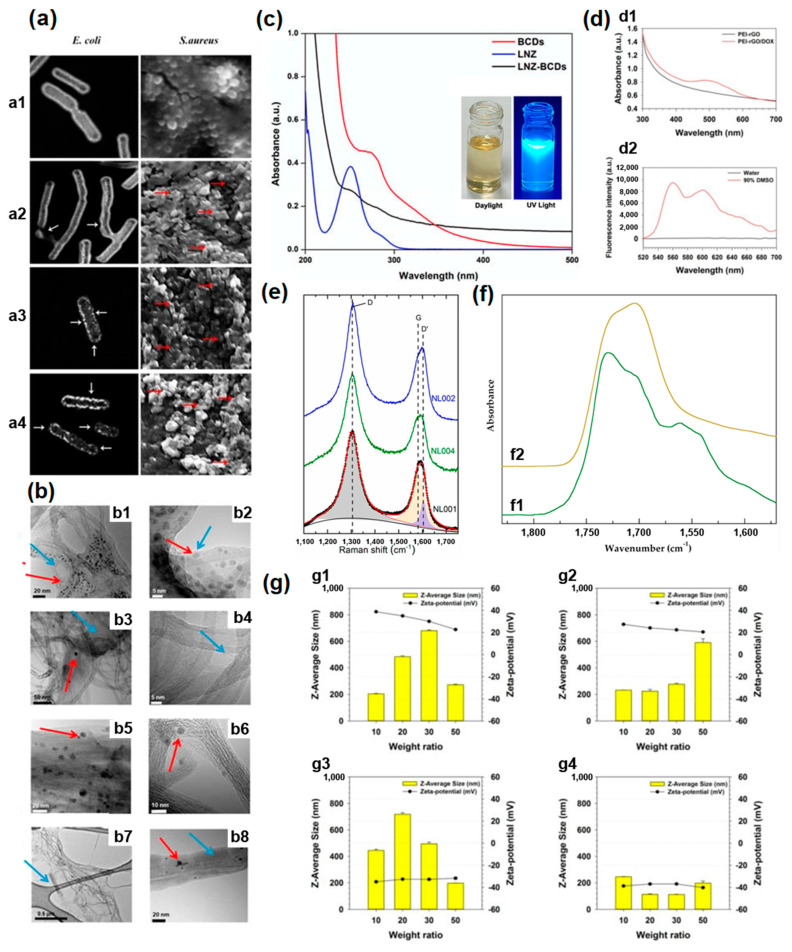
(**a**) (**a1**) The SEM images of non−treated bacterial cell. (**a2**) Bacterial cells treated with GONPs. (**a3**) Bacteria cells treated with GONPs–PEG. (**a4**) Bacterial cells exposed with GONPs–PEG–N. sativa. Left panels correspond to *E. coli*, and right panels belong to *S. aureus*. Copyright 2021 by the authors. Licensee MDPI, Basel, Switzerland. This article is an open−access article distributed under the terms and conditions of the Creative Commons Attribution (CC BY) license [56]. (**b**) Low, and high−magnification HRTEM images of MWCNT/PPGP_s_ (**b1**,**b2**) and MWCNT/PPGP_c_ (**b3**,**b4**), and SWCNT/PPGP_s_ (**b5**,**b6**) and SWCNT/PPGPc (**b7**,**b8**), accordingly. Copyright 2020 by the authors. Licensee MDPI, Basel, Switzerland. This article is an open−access article distributed under the terms and conditions of the Creative Commons Attribution (CC BY) license [41]. (**c**) (1) Ultraviolet (UV)−visible absorption spectra of synthetic oxazolidinone antibiotic linezolid (LNZ), bovine serum albumin carbon dots (BCDs), and LNZ–BCDs nanocomposite. (Inset) Photos of BCDs. Copyright 2023 by the authors. Licensee MDPI, Basel, Switzerland. This article is an open-access article distributed under the terms and conditions of the Creative Commons Attribution (CC BY) license [67]. (**d**) Absorbance spectra (**d1**), and fluorescence spectra (**d2**) of functionalized with poly(ethylene imine) (PEI) reduced GO, and PEI−rGO/DOX. Copyright 2019 by the authors. Licensee MDPI, Basel, Switzerland. This article is an open-access article distributed under the terms and conditions of the Creative Commons Attribution (CC BY) license [50]. (**e**) The Raman spectra of raw and functionalized magnetic multi−walled carbon nanotubes. Copyright 2020 by the authors. Licensee MDPI, Basel, Switzerland. This article is an open-access article distributed under the terms and conditions of the Creative Commons Attribution (CC BY) license [43]. (**f**) FT−IR spectra of the purified reduced carbon nanotube nanocomposite Nano 6 (**f1**) and the same sample at the end of hydrocortisone release (**f2**). Copyright 2020 by the authors. Licensee MDPI, Basel, Switzerland. This article is an open−access article distributed under the terms and conditions of the Creative Commons Attribution (CC BY) license [44]. (**g**) Z−Average sizes and zeta−potential values of (**g1**) PK_5_E_5_ (PEI−rGO), (**g2**) PK_5_E_7_ (PEI−rGO), (**g3**) PK_5_E_9_ (PEI−rGO), and (**g4**) PK_5_E_13_ (PEI−rGO) with various weight ratios (PKE: PEI−rGO). Copyright 2019 by the authors. Licensee MDPI, Basel, Switzerland. This article is an open−access article distributed under the terms and conditions of the Creative Commons Attribution (CC BY) license [50].

In order to observe the morphology of nanomaterials, different microscopies have been employed. Authors of Ref. [56] studied the effect of graphene oxide (GO) nanoparticles (GONPs), GONPs-polyethylene glycol (PEG) nanocomposite, and GONPs–PEG–N. sativa on the structure of organisms under treatment using the scanning electron microscopy (SEM) technique. Figure 1(a1) shows the scanning electron microscopy (SEM) images of non-treated bacterial cells [56]. Figure 1(a2) shows bacterial cells treated with GONPs. Figure 1(a3) shows bacteria cells treated with GONPs–PEG. Figure 1(a4) shows bacterial cell exposure with GONPs–PEG–N. sativa. The left panels correspond to *E. coli*, and the right panels belong to *S. aureus*.

SEM allows visualizing the effects. *E. coli* represent elongated-form bacteria, and all material influences its morphology. The modifications are denoted by white arrows in the left panels of the SEM images. *S. aureus* represent spherical-like material, whose modifications are marked by red arrows on the right panels. The modifications are observed for all material, and it is clearly visible on the SEM images. 

The images showed changes. The GONPs had moderate effects on the bacterial cell morphological changes. The GONPs-PEG nanocomposite damages the cell’s outer membrane. It is visible that the graphene oxide samples have severe effects on the morphology and lives of bacteria [56]. 

Figure 1b shows high-resolution transmission electron microscopy (HR-TEM) images for functionalized single-walled carbon nanotubes, and multi-walled carbon nanotubes (MWCNTs), their supramolecular adduct (CNT/pyrrole polypropylene glycol, PPGP_s_), and covalent adduct (CNT/PPGP_c_) [41]. The morphology of CNT/PPGP adducts is shown at different magnifications. Figure 1(b1–b4) shows MWCNT/PPGP_s_, and MWCNT/PPGP_c_, accordingly, and Figure 1(b5–b8) shows SWCNT/PPGP_s_ (C,c) and SWCNT/PPGPc (D,d), accordingly. 

### 3.2. Spectroscopy

#### 3.2.1. Optical Absorption Spectroscopy

The optical absorption spectroscopy of carbon nanomaterials for cytotoxic investigations was performed in Refs. [50,67]. In this section, we review the most interesting investigations. Authors of Ref. [67] investigated synthetic oxazolidinone antibiotic linezolid, BCDs nano-biocarrier, and LNZ–BCDs nanocomposite with optical absorption spectroscopy. Figure 1c shows the OAS spectra of LNZ, BCDs, and LNZ–BCDs nanocomposite. Figure 1c (inset) shows photos of BCDs. Figure 1(d1) shows the absorbance spectra of PEI-rGO and PEI-rGO/DOX [50]. Figure 1(d2) shows the fluorescence spectra of PEI-rGO/DOX. The characteristic peaks of DOX are observed [50]. 

#### 3.2.2. Raman Spectroscopy

Raman spectroscopy investigations of carbon nanomaterials for cytotoxic studies were performed in Refs. [43,75]. Figure 1e shows the Raman spectra of the NL001 sample that belongs to the sample pristine sample, the NL002 samples that are the pristine material functionalized by –COOH groups, and the NL004 sample that is functionalized by polyethyleneimine@Naproxen. 

#### 3.2.3. Fourier Transformed Infrared Spectroscopy

The FT-IR spectra of carbon nanomaterials for cytotoxic investigations were performed in Ref. [44]. Here, we review the most important examples. Figure 1f shows partial FT-IR spectra of the purified reduced carbon nanotube nanocomposite Nano 6 (Figure 1(f1)) and the same sample at the end of hydrocortisone release (Figure 1(f2)) [44].

### 3.3. Other Methods

Authors of Ref. [50] studied drug delivery by polymer-reduced graphene oxide composite. They plotted Z-Average sizes and zeta-potential values of (Figure 1(g1)) PK_5_E_5_ (poly(ethylene imine) (PEI)-rGO), (Figure 1(g2)) PK_5_E_7_(PEI-rGO), (Figure 1(g3)) PK_5_E_9_(PEI-rGO), and (Figure 1(g4)) PK_5_E_13_(PEI-rGO) with various weight ratios (PKE: PEI-rGO) [50]. 

## 4. Theoretical Methods

**Figure 2:**
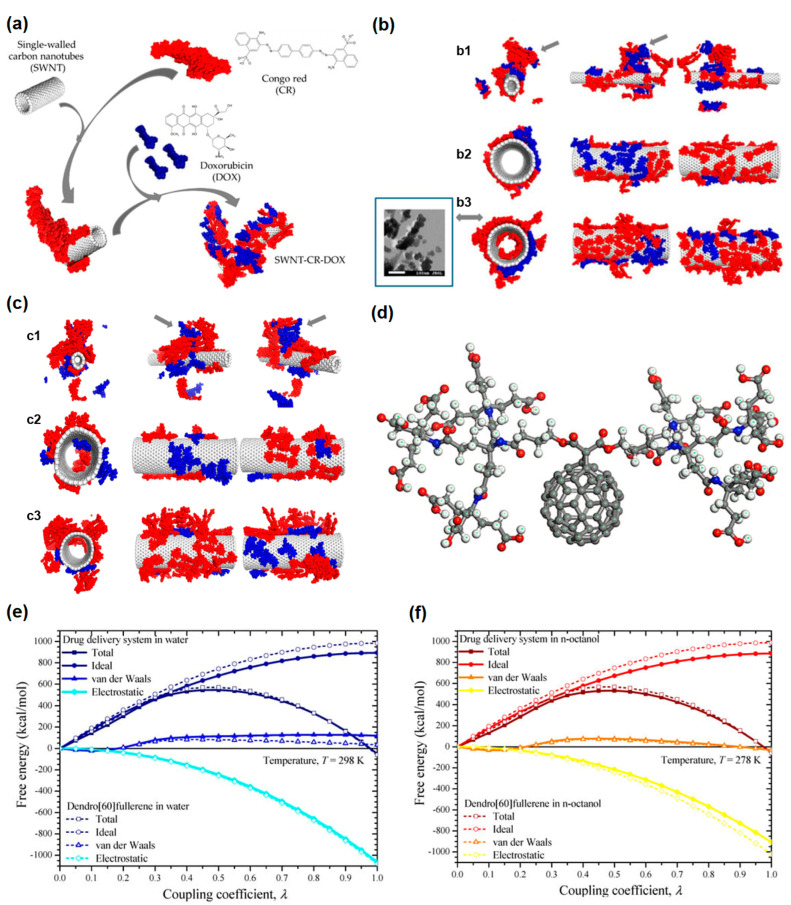
(**a**) The schematics of drug delivery system from SWCNTs, self−assembled ribbon−like structures (SRLS), Congo Red (CR) with drug doxorubicin. Copyright 2020 by the authors. Licensee MDPI, Basel, Switzerland. This article is an open−access article distributed under the terms and conditions of the Creative Commons Attribution (CC BY) license [42]. (**b**) The structure of SWCNT−CR-DOX systems at neutral pH values: (**b1**) SWCNT (10,0), 20 CR (red) and 10 DOX (blue), (**b2**) SWCNT (30,0), 20 CR and 10 DOX, (**b3**) SWCNT (30,0), 40 CR and 10 DOX. Inset: SEM image. Copyright 2020 by the authors. Licensee MDPI, Basel, Switzerland. This article is an open−access article distributed under the terms and conditions of the Creative Commons Attribution (CC BY) license [42]. (**c**) The structure of SWNT−CR−DOX systems at medium pH values (5.0 < pH < 7.4). (**c1**) SWCNT (10,0), 20 CR (red), 10 DOX (blue), (**c2**) SWCNT (30,0), 20 CR, and 10 DOX, (**c3**) SWCNT (30,0), 40 CR and 10 DOX. Copyright 2020 by the authors. Licensee MDPI, Basel, Switzerland. This article is an open−access article distributed under the terms and conditions of the Creative Commons Attribution (CC BY) license [42]. (**d**) The schematics of the dendro [60] fullerene. Copyright 2022 by the authors. Licensee MDPI, Basel, Switzerland. This article is an open−access article distributed under the terms and conditions of the Creative Commons Attribution (CC BY) license [63]. (**e**) Theoretical studies of functionalized drug−loaded fullerenes in water. Copyright 2022 by the authors. Licensee MDPI, Basel, Switzerland. This article is an open−access article distributed under the terms and conditions of the Creative Commons Attribution (CC BY) license [63]. (**f**) Theoretical studies of functionalized drug−loaded fullerenes in organic solvent. Copyright 2022 by the authors. Licensee MDPI, Basel, Switzerland. This article is an open−access article distributed under the terms and conditions of the Creative Commons Attribution (CC BY) license [63].

Authors of Ref. [42] developed the drug delivery system for the drug doxorubicin. This drug delivery system was capable of drug release under lower pH. Figure 2a shows the schematics of the drug delivery system [42]. 

Figure 2b shows the structure of SWCNT-CR-DOX systems at neutral pH values: SWCNT (10,0), 20 CR (red) and 10 DOX (blue) (Figure 2(b1)), SWCNT (30,0), 20 CR, and 10 DOX (Figure 2(b2)), SWCNT (30,0), 40 CR and 10 DOX (Figure 2(b3)). Inset: scanning electron microscopy (SEM) image [42]. Figure 2c shows the structure of SWNT-CR-DOX systems at medium pH values (5.0 < pH < 7.4), SWCNT (10,0), 20 CR (red), 10 DOX (blue) (Figure 2(c1)), SWCNT (30,0), 20 CR, and 10 DOX (Figure 2(c2)), SWCNT (30,0), 40 CR and 10 DOX (Figure 2(c3)) [42]. Part of DOX goes inside CNTs, as we see in Figure 2c. Authors of Ref. [63] theoretically studied the dendro [60] fullerene/molnupiravir drug delivery system (Figure 2d–f). 

## 5. Biological Effect of Nanomaterials upon Bacteria Cells In Vitro and In Vivo

### 5.1. Biological Effect of Nanomaterials upon Bacteria

**Figure 3:**
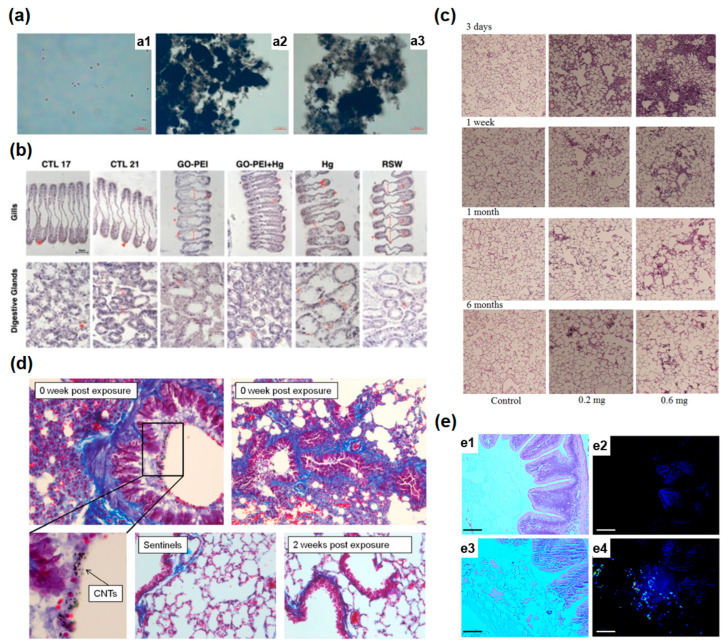
(**a**) (**a1**) Control; (**a2**) CNT-1; (**a3**) CNT-2 treated red algae P. purpureum. Copyright 2020 by the authors. Licensee MDPI, Basel, Switzerland. This article is an open-access article distributed under the terms and conditions of the Creative Commons Attribution (CC BY) license [7]. (**b**) Images of gills (upper), and digestive glands (lower) in Mytilus galloprovincialis influenced by different materials stained with hematoxylin. Scale bar = 50 µm. CTL: control, RSW: seawater after remediation (at 21 °C). Copyright 2021 by the authors. Licensee MDPI, Basel, Switzerland. This article is an open-access article distributed under the terms and conditions of the Creative Commons Attribution (CC BY) license [17]. (**c**) Hematoxylin–eosin (HE) staining of lung sections exposed to MWCNTs. Copyright 2012 by the authors. Licensee MDPI, Basel, Switzerland. This article is an open-access article distributed under the terms and conditions of the Creative Commons Attribution (CC BY) license [1]. (**d**) Animals exposed to CNTs and euthanized at 0 week post-exposure as well as 2 weeks post-exposure. Copyright 2014 by the authors. Licensee MDPI, Basel, Switzerland. This article is an open-access article distributed under the terms and conditions of the Creative Commons Attribution (CC BY) license [2]. (**e**) (**e1**,**e2**) show the representative light image and NIRF image of control sample of an intestinal cross-section of fish. (**e3**,**e4**) show representative light image and NIRF image of SWCNT fed fish. Scale bar = 150 μm. Copyright 2015 by the authors. Licensee MDPI, Basel, Switzerland. This article is an open-access article distributed under the terms and conditions of the Creative Commons Attribution (CC BY) license [5].

The visual observation of red algae P. purpureum after seven days of exposure to the nanoparticles of carbon nanotubes is presented in Figure 3a [7]. The agglomerates of carbon nanotubes are visible for two nanotube samples in Figure 3(a2,a3). 

Authors of Ref. [17] studied the influence of different materials on the gills and digestive glands of Mytilus galloprovincialis. For gills, Figure 3b (upper part) shows the histopathological alterations. The hemocyte infiltration (arrows), enlargement of the central vessel (long arrows), and abundance of lipofuscin aggregates (*) were observed, especially for Hg-treated samples [17]. For digestive glands, Figure 3b (lower part) shows the histopathological alterations. The hemocyte infiltration (arrows), atrophy, and necrosis were observed, especially in mussel tissue exposed to Hg (Figure 3b) [17]. 

### 5.2. Biological Effect of Nanomaterials upon Cell (In Vitro)

Hematoxylin–eosin (HE) staining of lung sections exposed to MWCNTs is presented in Figure 3c. The images of lung tissues after intratracheal instillation after 3 days, 1 week, 1 month, and 6 months for control samples of dosages of 0.2 mg and 0.6 mg are shown. Authors of Ref. [2] observed that animals exposed to CNTs and euthanized at 0 weeks post-exposure as well as 2 weeks post-exposure did not demonstrate major pathological changes (Figure 3d). Authors of Ref. [5] investigated the toxicity of SWCNTs for fish. SWCNTs can not be seen using light microscopy. Near-infrared fluorescence molecular imaging (NIRF) was used to detect the nanotubes in cross-sections. Bright fluorescence of SWCNTs was observed in treated tissues (Figure 3e) [5]. Figure 3(e1) shows the representative light image of the control sample. Figure 3(e2) shows the NIRF image of the control sample. Figure 3(e3,e4) shows the representative light image and NIRF image of SWCNT-fed fish. 

**Figure 4:**
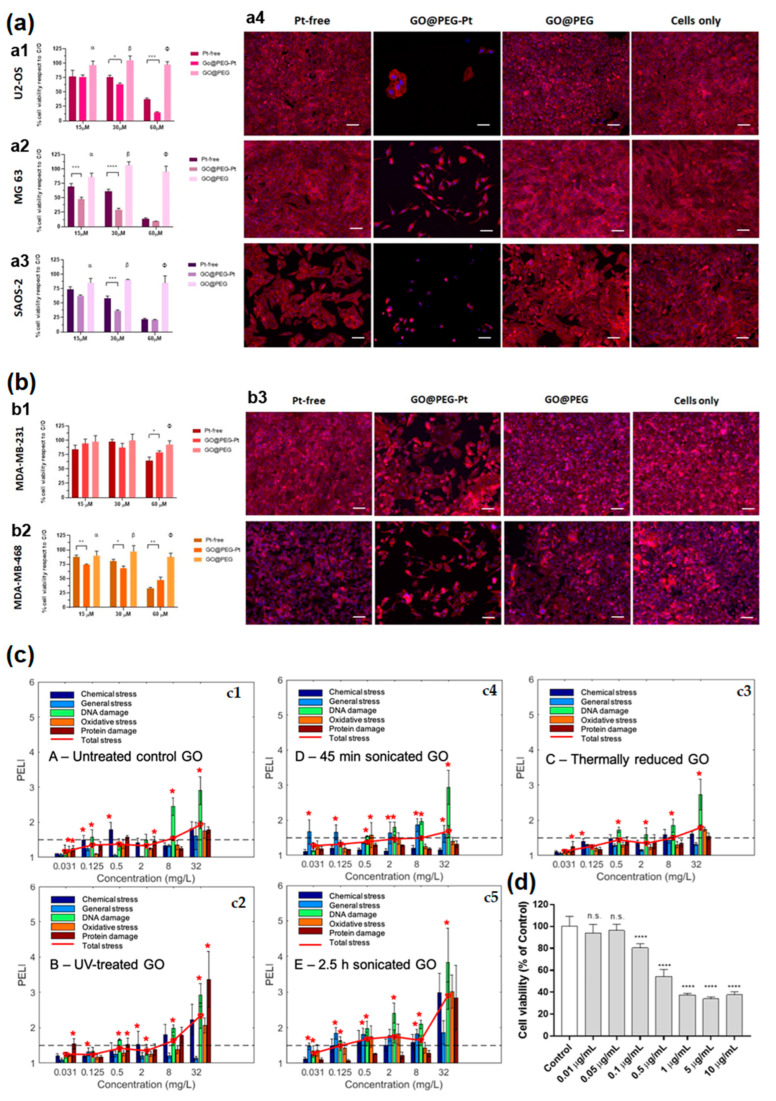
(**a**) The viability of U2-OS (**a1**), MG63 (**a2**), SAOS-2 cell lines (**a3**) and images of the cells cultured for 72 h in the presence of GO (**a4**). Scale bars, 100 μm. Copyright 2022 by the authors. Licensee MDPI, Basel, Switzerland. This article is an open-access article distributed under the terms and conditions of the Creative Commons Attribution (CC BY) license [60]. (**b**) The viability of U87 (**b1**), U118 (**b2**), and images of the cells cultured for 72 h in the presence of material (**b3**). Scale bars, 100 μm. Copyright 2022 by the authors. Licensee MDPI, Basel, Switzerland. This article is an open-access article distributed under the terms and conditions of the Creative Commons Attribution (CC BY) license [60]. (**c**) The comparison of the PELI values of different graphene samples, such as untreated graphene oxide (**c1**), UV treated graphene oxide (**c2**), thermally reduced graphene oxide (**c3**), 45 min sonicated graphene oxide (**c4**), 2.5 h sonicated graphene oxide (**c5**). The enrichment in toxicity is marked with *. Copyright 2021 by the authors. Licensee MDPI, Basel, Switzerland. This article is an open-access article distributed under the terms and conditions of the Creative Commons Attribution (CC BY) license [19]. (**d**) The cytotoxicity of graphene nanoparticles in human keratinocytes cells (HaCaT) for exposure period of 24 h and concentrations from 0 (control) to 10 µg/mL measured by the (3-(4,5-dimethylthiazol-2-yl)-2,5-diphenyl tetrazolium) (MTT) assay. Copyright 2022 by the authors. Licensee MDPI, Basel, Switzerland. This article is an open-access article distributed under the terms and conditions of the Creative Commons Attribution (CC BY) license [21]. * *p*-value < 0.05, ** *p*-value < 0.01,*** *p*-value < 0.001, **** *p*-value < 0.0001, α, β, Φ: the mean ± standard error of the mean, n.s: not significant.

Authors of Ref. [60] studied the cell viability in osteosarcoma cell lines U2-OS (Figure 4(a1)), MG63 (Figure 4(a2)), SAOS-2 (Figure 4(a3)) in graphene oxide@polyethylene glycol (PEG). An (3-(4, 5-dimethylthiazol-2-yl)-2, 5-diphenyl tetrazolium) (MTT) assay was performed after 72 h of cell culture (Figure 4(a4)) [60]. 

Authors of Ref. [60] studied the cell viability in osteosarcoma cell lines U87 (Figure 4(b1)) and U118 (Figure 4(b2)) in graphene oxide@PEG. MTT assay was made after 72 h of glioblastoma cell line (Figure 4(b3)) [60]. The protein expression level index (PELI) values in stress conditions for different concentrations of graphene oxide samples are shown in Figure 4c [19]. It is visible that in specific conditions, there is an enhancement of toxicity (asterisk, Figure 4c). In Ref. [21], the cytotoxicity of graphene nanoparticles in human keratinocytes cells (HaCaT) for an exposure period of 24 h and concentrations from 0 (control) to 10 µg/mL were analyzed. It is visible that there is no cytotoxicity for concentrations below ≤0.05 µg/mL (Figure 4d).

**Figure 5:**
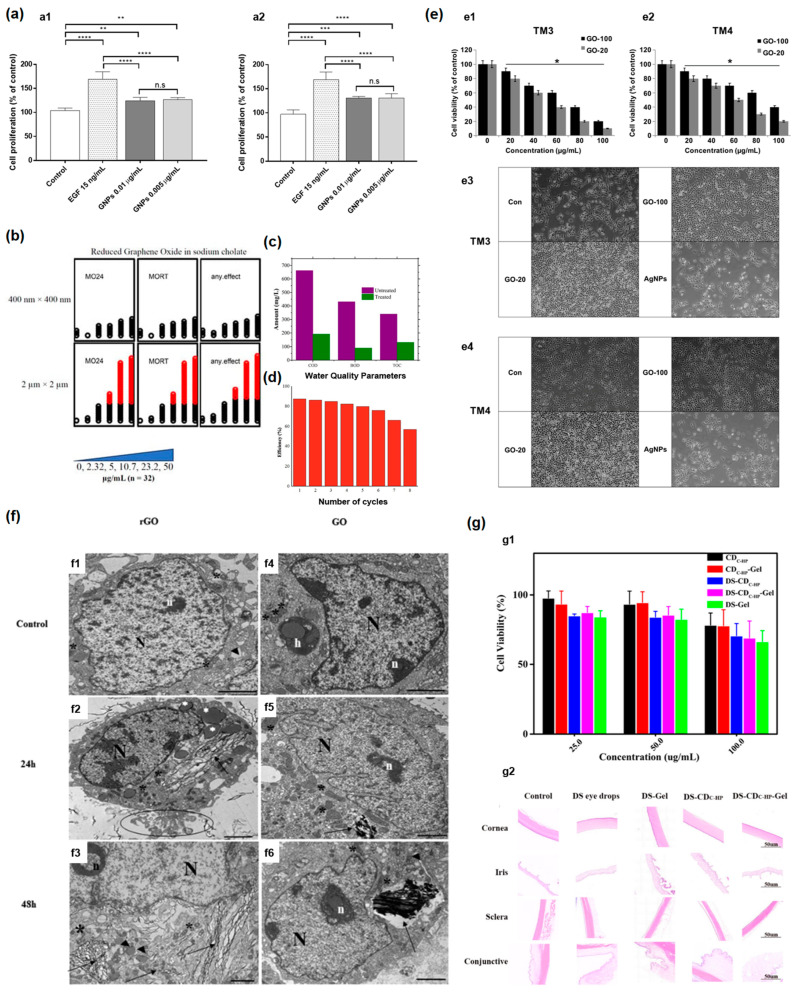
(**a**) The proliferative activity of graphene nanoparticles in the HaCaT cell was investigated at 0.005 and 0.01 μg/mL for 72 (**a1**) or 96 h (**a2**). The enrichment in toxicity is marked with *. Copyright 2022 by the authors. Licensee MDPI, Basel, Switzerland. This article is an open-access article distributed under the terms and conditions of the Creative Commons Attribution (CC BY) license [21]. (**b**) Exposure of embryos (*n* = 32) to reduced graphene oxide revealed that for the 2 μm × 2 μm reduced graphene oxide, there was significant mortality for concentrations starting 10.7 μg/mL, whereas 400 nm × 400 nm reduced graphene oxide did not lead to significant mortality at tested concentrations. MO24 = 24 post-fertilization (hpf) mortality; MORT = 120 hpf mortality. Copyright 2022 by the authors. Licensee MDPI, Basel, Switzerland. This article is an open-access article distributed under the terms and conditions of the Creative Commons Attribution (CC BY) license [22]. (**c**) The waste water quality parameters, biological oxygen demand (BOD), chemical oxygen demand (COD), and total organic carbon (TOC) before and after photocatalytic treatment with aerogel photocatalytic membrane based on graphene oxide (GO) with Cr–Mn-doped TiO_2_. Copyright 2022 by the authors. Licensee MDPI, Basel, Switzerland. This article is an open-access article distributed under the terms and conditions of the Creative Commons Attribution (CC BY) license [26]. (**d**) The dependence of efficiency of Cr–Mn-doped TiO_2_@GO aerogel photocatalytic membranes on number of cycles. Copyright 2022 by the authors. Licensee MDPI, Basel, Switzerland. This article is an open-access article distributed under the terms and conditions of the Creative Commons Attribution (CC BY) license [26]. (**e**) (**e1**,**e2**) Cell viability of TM3, and TM4 cells treated with graphene oxide samples with the average size of 20 nm (GO-20), and 100 nm (GO-100). (**e3**,**e4**) TM3, and TM4 cell morphology, respectively, under a light microscope, compared with control sample (Con), and silver nanoparticles (AgNPs) (scale bar 200 µm). The enrichment in toxicity is marked with *. Copyright 2019 by the authors. Licensee MDPI, Basel, Switzerland. This article is an open-access article distributed under the terms and conditions of the Creative Commons Attribution (CC BY) license [155]. (**f**) TEM images of cellular internalization of reduced graphene oxide (rGO) and graphene oxide (GO) in Caco-2 cells. (**f1**,**f4**) untereated control sample. (**f2**,**f3**) Caco-2 treated with rGO after 24 h and 48 h, respectively. (**f5**,**f6**) Caco-2 treated with GO after 24 h and 48 h, respectively (scale bar: 2 μm). Signs are h—heterophagosome; N—nucleus; *n*—nucleolus; *—mitochondria; ▴—endoplasmic reticulum; ì—dense bodies; apoptotic bodies (circle) and graphene materials (black arrows). Copyright 2022 The Authors. Published by Elsevier B.V. This is an open-access article under the CC BY license [156]. (**g**) (**g1**) Cell viability for different concentrations of samples carbon dots, carbon dots-gel, DS-carbon dots, DS-carbon dots-Gel, DS-Gel for 24 h. (**g2**) Histopathological microscopy of ocular tissues. Copyright 2021 by the authors. Licensee MDPI, Basel, Switzerland. This article is an open-access article distributed under the terms and conditions of the Creative Commons Attribution (CC BY) license [64]. * *p*-value < 0.05, ** *p*-value < 0.01,*** *p*-value < 0.001, **** *p*-value < 0.0001, n.s: not significant.

The proliferative activity of graphene nanoparticles in the HaCaT cell was investigated at 0.005 and 0.01 μg/mL for 72 or 96 h (Figure 5(a1,a2)) [21]. 

Authors of Ref. [22] investigated the developmental toxicity of reduced graphene oxide. It was shown that 2 μm × 2 μm reduced graphene oxide caused higher embryonic mortality than the sample of 400 nm × 400 nm reduced graphene oxide. For the 2 μm × 2 μm reduced graphene oxide, there was significant mortality for concentrations starting at 10.7 μg/mL, whereas 400 nm × 400 nm reduced graphene oxide did not lead to significant mortality at tested concentrations (Figure 5b). Authors of Ref. [26] compared the wastewater quality parameters, biological oxygen demand (BOD), chemical oxygen demand (COD), and total organic carbon (TOC) before and after photocatalytic treatment with aerogel photocatalytic membrane based on graphene oxide with Cr–Mn-doped TiO_2_ (Figure 5c) [26]. Figure 5d shows the dependence of the efficiency of membranes on the number of cycles [26]. It is visible that the efficiency is retained and then decreases with increasing the number of cycles. Even after eight cycles, the membrane kept its efficiency of more than 50% in the wastewater. 

Graphene oxide cytotoxicity is dependent on flake size and oxygen group composition. In Ref. [155], the authors studied the dependence of cytotoxicity on graphene flake size. Two samples with an average size of 20 nm (GO-20) and 100 nm (GO-100) were studied. It was shown that both samples inhibit the viability of TM3 and TM4 cells. However, with 100 nm-size graphene flakes, the cell viability is higher (Figure 5(e1,e2)). Figure 5(e3,e4) shows TM3, and TM4 cell morphology, respectively, under a light microscope, compared with the control sample (Con) and silver nanoparticles (AgNPs) (scale bar 200 µm) [155]. 

In Ref. [156], the authors studied the dependence of cytotoxicity on oxygen group composition. Figure 5f shows TEM images of cellular internalization of reduced graphene oxide (rGO) and graphene oxide (GO) in Caco-2 cells. Figure 5(f1,f4) shows an untreated control sample. Figure 5(f2,f3) shows Caco-2 treated with rGO after 24 h and 48 h, respectively. Figure 5(f5,f6) shows Caco-2 treated with GO after 24 h and 48 h, respectively (scale bar: 2 μm). They found that cells treated with rGO showed significant changes, whereas cells treated with GO did not show such modifications. Cells treated with rGO for 24 h (Figure 5(f2)) demonstrated improved heterochromatin in the nucleus. Dense bodies in the cytoplasm, as well as cell fragmentation, were observed. There is rGO inside cells. Cells treated with GO for 24 h (Figure 5(f5)) heterochromatic nuclei were detected. Mitochondria are better conserved than rGO. There is GO inside Caco-2 cells, too. Cells treated with rGO for 48 h (Figure 5(f3)) exhibited the segregation of the nucleolus. The endoplasmatic reticulum appears as a dense body. Cells treated with GO for 48 h (Figure 5(f6)) showed more modified mitochondria than after 24 h [156]. 

Figure 5(g1) shows in vitro cytotoxicity of different samples of carbon dots, carbon dots-gel, diclofenac sodium (DS)-carbon dots, DS-carbon dots-Gel, and DS-Gel. Figure 5(g2) shows histopathological microscopy of treated ocular tissues [64].

### 5.3. Biological Effect of Nano Materials upon In Vivo

**Figure 6:**
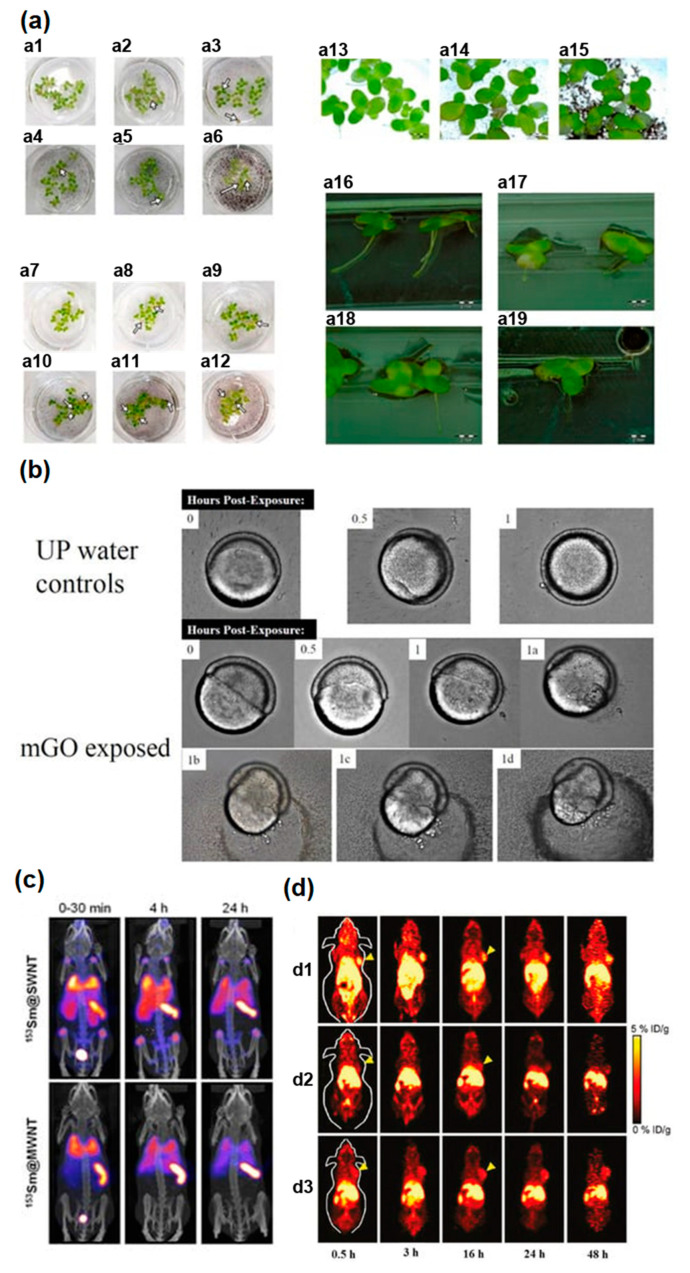
(**a**) Lemna minor morphology after influence of pristine graphene and graphene oxide. (**1**) Control group. (**a1**–**a6,a13,a14**,**a16,a17**) after influence of pristine graphene. (**a7**–**a12**,**a15**,**a18**,**a19**) after graphene oxide influence. Copyright 2021 by the authors. Licensee MDPI, Basel, Switzerland. This article is an open-access article distributed under the terms and conditions of the Creative Commons Attribution (CC BY) license [30]. (**b**) Brightfield imaging of 6 hpf embryos for different post-exposure periods, in comparison with exposures to ultrapure water controls. Copyright 2022 by the authors. Licensee MDPI, Basel, Switzerland. This article is an open-access article distributed under the terms and conditions of the Creative Commons Attribution (CC BY) license [22]. (**c**) The images of a whole-body single-photon emission computed tomography/computed tomography (SPECT/CT) imaging, and quantitative γ-counting of tissue biodistribution of ^153^Sm@SWCNTs and ^153^Sm@MWCNTs. Reprinted with permission from [76]. Copyright 2020 American Chemical Society. (**d**) Targeting ability of ^64^Cu-NOTA-GO-TRC105 towards 4T1 tumor-bearing mice (NOTA = a chelating agent of ^64^Cu). (**d1**) The post injected PET images. (**d2**) ^64^Cu-NOTA-GO alone TRC105. (**d3**) Preinjected blocking dose of TRC105. Reprinted with permission from Ref. [94]. Copyright 2012 American Chemical Society.

Authors of Ref. [30] studied the toxicity of graphene and GO on plant Lemna minor. It was observed that there was a decrease in the size of the leaves (white arrows, Figure 6a), and the roots had an appearance of white highlights. This was observed for graphene and GO. 

Figure 6b shows brightfield imaging of 6 hpf embryos exposed to 400 nm × 400 nm reduced graphene oxide for different post-exposure periods in comparison with exposures to ultrapure water controls [22]. In Ref. [76], to study pharmokinetics, and biodistribution of ^153^Sm@SWCNTs and ^153^Sm@MWCNTs, bioimaging, a whole-body single-photon emission computed tomography/computed tomography (SPECT/CT) imaging, and quantitative γ-counting were applied The accumulation in the spleen, lung, and liver within 30 min was observed, as it is shown in Figure 6c. Positron emission tomography (PET), CT, with the functionalization of radioactive elements, was applied [94]. In Ref. [94], ^64^Cu was linked with GO-PEG via 1,4,7-triazacyclo nonane-1,4,7 triacetic acid (NOTA, a chelating agent of ^64^Cu). ^64^Cu-NOTA-GO conjugated with TRC105 (^64^Cu-NOTA-GO-TRC105) for targeting a CD105 (endogline) was synthesized. Figure 6d shows its targeting ability toward 4T1 tumor-bearing mice [94].

## 6. Different Biological Effects on Drug and Nanomaterial + Drug

### 6.1. Drug Toxicity and Nanomaterial + Drug Toxicity

**Figure 7:**
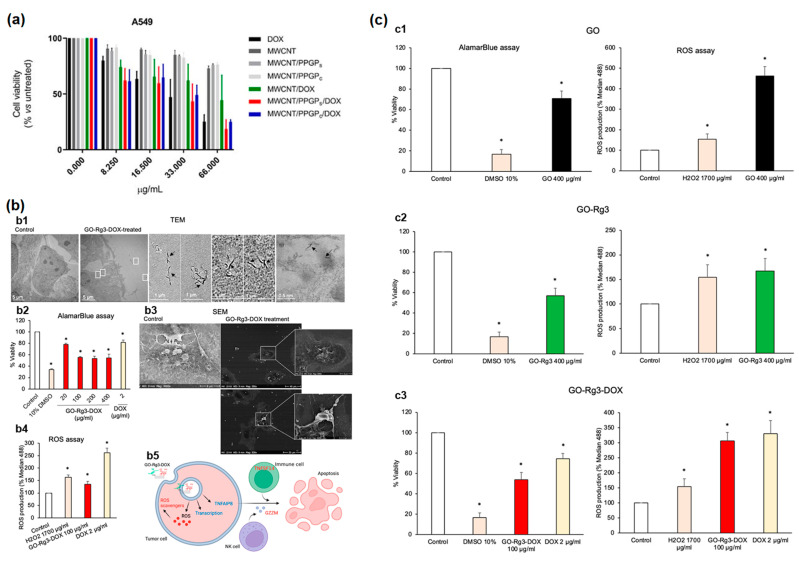
(**a**) The cell viability (MTT assay) for different concentrations of unloaded DOX, MWCNTs, nanocomposites, loaded DOX against A549 human melanoma cells after 48 h of treatment. Copyright 2020 by the authors. Licensee MDPI, Basel, Switzerland. This article is an open-access article distributed under the terms and conditions of the Creative Commons Attribution (CC BY) license [41]. (**b**) (**b1**) TEM images of treated cells. (**b2**) AlamarBlue cell viability assay 24 h after treatment with GO–Rg3–DOX. (**b3**) SEM of treated cells. (**b4**) Reactive oxygen species (ROS). (**b5**) Schematics of GO–Rg3–DOX internalization. The enrichment in toxicity is marked with *. Copyright 2023 by the authors. Licensee MDPI, Basel, Switzerland. This article is an open-access article distributed under the terms and conditions of the Creative Commons Attribution (CC BY) license [27]. (**c**) Rg3, DOX, graphene oxide cytotoxicity in human breast cancer MDA-MB-231 cells. AlamarBlue assay 24 h after GO (**c1**), GO–Rg3 (**c2**), GO–Rg3–DOX (**c3**) treatment is shown together with ROS production (on the right side). The enrichment in toxicity is marked with *. Copyright 2023 by the authors. Licensee MDPI, Basel, Switzerland. This article is an open-access article distributed under the terms and conditions of the Creative Commons Attribution (CC BY) license [27].

Authors of Ref. [41] studied the cell viability (an (3-(4,5-dimethylthiazol-2-yl)-2,5-diphenyl tetrazolium) (MTT) assay) for different concentrations of unloaded DOX, MWCNTs, nanocomposites, loaded DOX against A549 human melanoma cells (Figure 7a) after 48 h of treatment. The chosen concentrations were 8.25 µg/mL, 16.5 µg/mL, 33 µg/mL, 66 µg/mL. The harmful effect of DOX was maintained for all investigated samples on both cells. 

Authors of Ref. [27] performed the studies of cytotoxicity of graphene oxide and GO– Ginsenoside (Rg3)– Doxorubicin against Huh7 cells. Figure 7(b1) shows the transmission electron microscopy images of treated cells. Figure 7(b2) shows AlamarBlue cell viability assay 24 h after treatment with GO–Rg3–DOX. Figure 7(b3) shows the scanning electron microscopy of treated cells. Figure 7(b4) shows reactive oxygen species (ROS). Figure 7(b5) shows schematics of GO–Rg3–DOX internalization [27]. 

Figure 7c shows Rg3, DOX, and graphene oxide cytotoxicity in human breast cancer MDA-MB-231 cells [27]. AlamarBlue assay 24 h after GO, GO–Rg3, GO–Rg3–DOX treatment is shown together with ROS production. The Rg3 component reduced ROS generation. Therefore, it reduced the side effects on non-cancerous tissues. 

### 6.2. Synergy or Antagonistic Effect

**Figure 8:**
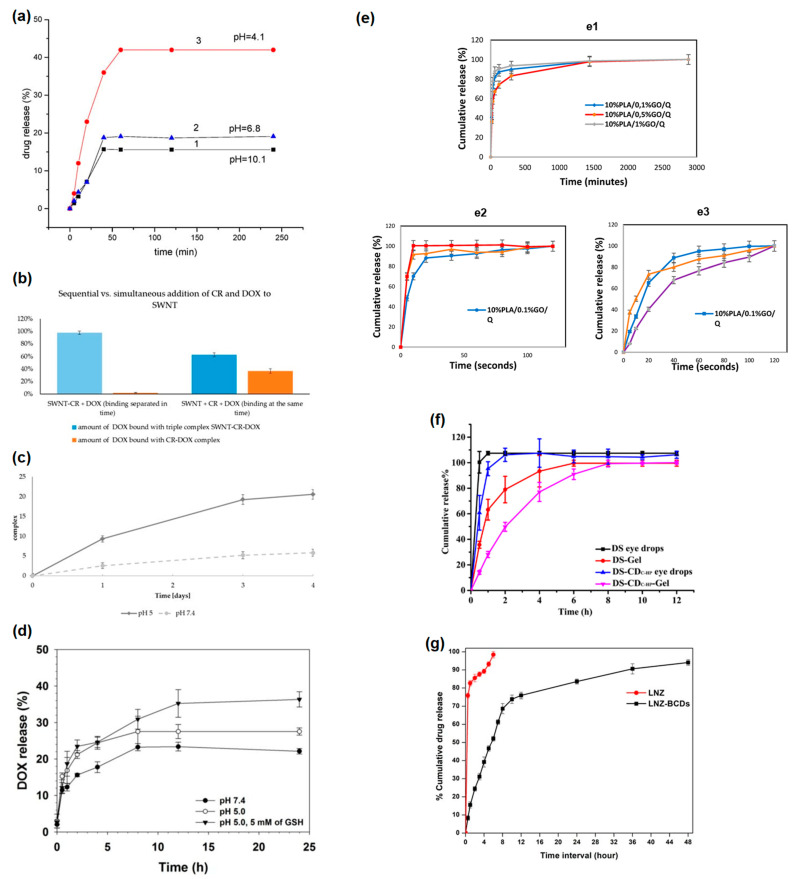
(**a**) The drug release from the functionalized carbon nanotubes at different pH assessed by fluorescence measurements under excitation at 259 nm. Copyright 2020 by the authors. Licensee MDPI, Basel, Switzerland. This article is an open-access article distributed under the terms and conditions of the Creative Commons Attribution (CC BY) license [43]. (**b**) Sequential versus simultaneous addition of CR and DOX to SWNT. Percent of DOX added to the sample found in complexes. Congo Red (CR), anticancer drug doxorubicin. Copyright 2019 by the authors. Licensee MDPI, Basel, Switzerland. This article is an open-access article distributed under the terms and conditions of the Creative Commons Attribution (CC BY) license [40]. (**c**) DOX release from SWCNT-CR-DOX complex at pH 5, and pH 7.4. Copyright 2019 by the authors. Licensee MDPI, Basel, Switzerland. This article is an open-access article distributed under the terms and conditions of the Creative Commons Attribution (CC BY) license [40]. (**d**) The dependence of DOX release on time PK_5_E_7_ (PEI-rGO/DOX) at different pH. Copyright 2019 by the authors. Licensee MDPI, Basel, Switzerland. This article is an open-access article distributed under the terms and conditions of the Creative Commons Attribution (CC BY) license [50]. (**e**) Cumulative release of Quercetin from the microfiber scaffolds without electric stimulus (**e1**), and under 10 Hz (**e2**), 50 Hz (**e3**). Copyright 2021 by the authors. Licensee MDPI, Basel, Switzerland. This article is an open-access article distributed under the terms and conditions of the Creative Commons Attribution (CC BY) license [52]. (**f**) In vitro release profiles. Copyright 2021 by the authors. Licensee MDPI, Basel, Switzerland. This article is an open-access article distributed under the terms and conditions of the Creative Commons Attribution (CC BY) license [64]. (**g**) The comparison of in vitro release profile of the LNZ, LNZ–BCDs nanocomposite. Copyright 2023 by the authors. Licensee MDPI, Basel, Switzerland. This article is an open-access article distributed under the terms and conditions of the Creative Commons Attribution (CC BY) license [67].

Figure 8a shows the drug release from the PEI-functionalized carbon nanotubes at different pH [43]. It is visible that the release of drugs is maximal at pH 4.1. Other pH leads to much lower releases (19% and 16%, accordingly).

Authors of Ref. [40] studied sequential versus simultaneous addition of Congo Red and anticancer drug doxorubicin to single-walled carbon nanotubes. In Figure 8b, it is visible that the amount of DOX bound with triple complex SWCNT-CR-DOX is larger than the amount of DOX bound with CR-DOX complex. Figure 8c shows DOX release from the SWCNT-CR-DOX complex at pH 5 and pH 7.4 at room temperature [40]. It is visible that there is a gradual release of DOX. The values are obtained by the spectrofluorimetrical method. DOX release is better at lower pH because it is an acidic media. Figure 8d shows the dependence of DOX release on time PK_5_E_7_ (PEI-rGO/DOX) at different pH [50]. There is an easy release of DOX in acidic conditions. 

### 6.3. Advantages and Disadvantages of Using Nanomaterials

Authors of Ref. [52] studied the cumulative release of Quercetin from the microfiber scaffolds without electric stimulus (Figure 8(e1)), and under 10 Hz (Figure 8(e2)), 50 Hz (Figure 8(e3)). Figure 8f shows in vitro release profiles. Figure 8g shows the comparison of in vitro release profile of the LNZ, LNZ–BCDs nanocomposite [67]. It is visible that there is the release of almost 98 ± 0.5% after 6 h of LNZ from the free drug LNZ solution. The release of LNZ–BCDs nanocomposite has a similar manner. It is visible that the release of LNZ does not exceed 52 ± 1.3% after 6 h from LNZ–BCDs nanocomposite. This system has the following advantages and disadvantages:

- BCDs have a small size, suitable optical and photoluminescence properties, and good photostability, and that is why they are promising nanocarriers of LNZ,
- LNZ–BCDs nanocomposites show biphase release, which is important for improving tissue healing,
- LNZ–BCDs nanocomposites were shown to have good biocompatibility and low cytotoxicity for human cells,
- LNZ–BCDs nanocomposites have good antibacterial properties,
- LNZ–BCDs nanocomposites have increased cell proliferation, which improves tissue regeneration and healing effect,
- LNZ–BCDs nanocomposites can be considered as a replacement for toxic nanoparticles in biomedical applications and for drug delivery to mend humans [67].

## 7. Perspectives

We discussed in Section 2 that there are three cytotoxicity issues: material characterization, theoretical considerations, cell viability, and drug release. 

Materials characterization methods are developed, and the progress of this topic is dependent on the achievements of microscopy, spectroscopy, and other methods, such as zeta potential measurements. The increase in accuracy and resolution of characterization techniques are significant, and it is connected with progress in synthesis methods of carbon nanomaterials, and theoretical considerations help. 

Cell viability is important to be improved here. Cell viability can be used to investigate chemicals for harmful influence on different cells. It can be used to choose anticancer drugs. It is important to improve the detection sensitivity and experiments. This leads to improved knowledge about the cytotoxicity of chemicals and carbon nanomaterials on tissues, organs, and the whole body. 

The important issue is single-cell viability. It can allow a correlation of the functionality of organelles of single cells with the development of cancer. Such organelles are mitochondria, nuclei, vacuoles, and reticulum. It allows for finding biochemical evidence of cancer prevention in single cells. This means improving the time of development of the disease and the time of treatment of the disease. 

The development of investigation methods for single-cell level detection of viability stimulates the development of the cytotoxicity investigative field. The development of methods of single-cell microscopy is needed to improve the resolution and accuracy of investigations, and the influence of chemicals on single cells is important to study here. 

The important for cytotoxicity is drug release. It is important to control the amount of drug that is released. This is performed with pH, temperature, and electric stimulation. The further development of methods of drug loading and bioimaging is important to decrease the cytotoxicity of carbon nanomaterials. The delivery of both biomedical contrast agents and therapeutic drugs using carbon nanomaterials is required. 

## 8. Conclusions

In this review, we considered the issues of cytotoxicity of carbon nanomaterials. We performed detailed studies on the topics and revealed that such issues of cytotoxicity as solubility and cellular uptake could be overcome with the chemical functionalization of carbon nanomaterials. These can be performed by covalence, noncovalence, intercalation, substitution, and filling. The future work is dedicated to the chemical functionalization of carbon nanomaterials toward applications, in particular, in the biomedical field, nanoelectronics, bioelectronics, biosensors, light emission, biolight emission, bioelectronics light emission, thermoelectric power generation, solar cells, catalysts, spintronics, drug delivery, and bioimaging for plants. Our next review is dedicated to drug delivery, bioimaging of carbon nanomaterials, and biosensors for plants, microorganisms, animals, and humans. 

## Data Availability

The data are available on request from the first author.

## Nomenclature

BCD	bovine serum albumin carbon dots
BOD	biological oxygen demand
CD	carbon dot
CNT	carbon nanotube
COD	chemical oxygen demand
CR	Congo Red
CT	computer tomograhy
DOX	doxorubicin
DS	diclofenac sodium
FT-IR	Fourier transformed infrared
GO	graphene oxid
HE	Hematoxylin–eosin
LNZ	linezolid
MWCNT	multi-walled carbon nanotubes
NOTA	1,4,7-triazacyclo nonane-1,4,7 triacetic acid
PEG	polyethylene glycol
PEI	poly(ethylene imine)
PELI	protein expression level index
PET	positron emission tomography
Q	Quercitine
rGO	reduced graphene oxide
ROS	reactive oxygen species
SEM	scanning electron microscopy
SPECT	single-photon emission computed tomography
SRLS	self-assembled ribbon-like structures
SWCNT	single-walled carbon nanotube
TEM	transmission electron microscopy
TOC	total organic carbon
